# The COVID-19 Crisis in Sub-Saharan Africa: Knowledge, Attitudes, and Practices of the Nigerian Public

**DOI:** 10.4269/ajtmh.20-0461

**Published:** 2020-09-22

**Authors:** Oluwaseyitan A. Adesegun, Tolulope Binuyo, Oluwafunmilola Adeyemi, Osaze Ehioghae, David F. Rabor, Oyebola Amusan, Olutosin Akinboboye, Omiete F. Duke, Ayobami G. Olafimihan, Oluwafemi Ajose, Akolade O. Idowu, Olumide Abiodun

**Affiliations:** 1 Benjamin S. Carson (Snr.) College of Medicine, Babcock University, Ilishan-Remo, Ogun State, Nigeria;; 2 Ministry of Health, Ibadan, Oyo State, Nigeria;; 3 College of Medicine, University of Ibadan, Ibadan, Oyo State, Nigeria;; 4 Department of Internal Medicine, Babcock University Teaching Hospital, Ilishan-Remo, Nigeria;; 5 General Hospital, Odan, Lagos State, Nigeria;; 6 University of Ilorin Teaching Hospital, Ilorin, Nigeria;; 7 School of Public Health, University of South Wales, Rhondda Cynon Taff, United Kingdom;; 8 Department of Community Medicine, Babcock University Teaching Hospital, Ilishan-Remo, Ogun State, Nigeria

## Abstract

Within a short period of time, COVID-19 has spread globally, wreaking havoc in various facets of life. This study sought to measure the level of COVID-19 knowledge, attitudes, and practices of the Nigerian public. This was a cross-sectional online survey of the general population of educated Nigerians who had Internet access. Sociodemographic data and participants’ knowledge, attitudes, and practices relating to COVID-19 were collected. Scores assessing knowledge, attitudes, and practices were allocated and graded based on specific stratified demarcations. Student’s *t*-test, analysis of variance, and logistic regression analysis were used where appropriate. Of the total 1,015 respondents, most of them exhibited good knowledge of COVID-19, with a mean knowledge grade of 78%; this significantly affected their attitude and practice grades (66% and 60.4%, respectively). Most respondents expressed positive attitudes toward foreigners and other stigma-prone groups, while also practicing appropriate preventive measures. Those aged 21–30 years and those with medical-related occupations had significantly higher knowledge scores (*P* < 0.001); and having a medical-related occupation increased the likelihood of having good knowledge compared with being unemployed (odds ratio [95% CI]: 6.6 [2.5–17.3]). Male participants aged 21–30 years and those with medical-related occupations had significantly higher attitude scores (*P* < 0.05). Engaging literate Nigerians on various media platforms, particularly social media, will result in wider reach for the purpose of COVID-19 education. Further studies on other sociodemographic groups within the country (e.g., the less educated) would give a clearer picture of the Nigerian situation as regards COVID-19 knowledge, attitudes, and practices (coronavirus, COVID-19, Public health, Nigeria, Africa).

## INTRODUCTION

Coronaviruses are a diverse family of RNA viruses that can infect a variety of hosts, causing disease states that range from common cold to more fatal diseases such as SARS and Middle East respiratory syndrome in humans. Although these viruses have been described for more than 50 years, the index outbreak was caused by a new strain, SARS-CoV-2.^1,2^ COVID-19 often presents with a respiratory syndrome that ranges in severity from dry cough, malaise, fever, sore throat, and new loss of taste/smell to difficulty in breathing and respiratory distress, with features of hypoxia in severe cases.^3^ Reports have also included vomiting and diarrhea in the symptomatology of the disease.^3,4^ COVID-19 has, within a short time, spread across the world, with the WHO declaring the outbreak a public health emergency of international concern on January 30, 2020.^5^

Nigeria is a country of concern in Africa with respect to this pandemic, owing to its dense population of more than 200 million people, 24.6 million of whom live in Lagos, the commercial capital of the country and the initial epicenter of the COVID-19 crisis in Nigeria.^6–8^ The first confirmed case in Nigeria was an Italian man who returned from Milan, Italy, to Lagos, Nigeria, on February 27, 2020.^9^ Case detection has progressively risen since the first case, and as at June 27, 2020, there were a total of 23,298 confirmed cases and 554 deaths.^8^

The Nigeria CDC (NCDC) is the nation’s public health institute, which deals with preparedness, detection, and response to infectious disease outbreaks and public health emergencies.^10^ The Presidential Task Force on COVID-19 was established by President Mohammadu Buhari on March 9, 2020, to coordinate the country’s multi-sectoral, intergovernmental efforts to contain the spread and mitigate the impact of the COVID-19 pandemic in Nigeria.^11^ The Federal Ministry of Health through the NCDC has activated a national emergency operations centre (EOC), which leads the national public health response to the COVID-19 outbreak in Nigeria, along with state EOCs leading efforts for the states. The NCDC also works in close connection with the state governments of all affected states through the deployment of national rapid response teams to support response activities, including contact tracing.^8^ Some of the measures instituted by the federal government of Nigeria include issuing a ban on all international flights effective from March 23, 2020, except for emergency and essential flights. On March 30, 2020, the federal government also imposed a lockdown of non-essential activities in the Federal Capital Territory, Abuja, Lagos, and Ogun states. The NCDC continues to expand laboratories for the testing of COVID-19. As at the time of this study, there were 29 laboratories in the NCDC’s molecular laboratory network, equipped to test for COVID-19 in Nigeria.^8^

Social stigma in the context of health, is when a negative association is made between a person or group of people who share certain characteristics and a specific disease. In the context of this outbreak, this may mean people are labeled, stereotyped, discriminated against, treated separately, and/or experience loss of status because of a perceived link with the disease.^12^ There have been multiple reports from the United States, of foreigners particularly from China and other Asian countries, being subjects of intense scrutiny based solely on the misinformation and stigmatizing attitudes of the public.^13–15^ The story is no different in Nigeria, as people of Asian ethnicity and recent international travelers in Nigeria may be perceived as the cause of the pandemic reaching the shores of Nigeria. Some researchers suggest that enforcing travel restrictions potentiates racial/ethnic stigmatization by restricting global cooperation, encouraging verbal and physical abuse against certain foreign nationals and ethnicities (particularly people of Asian descent) and discouraging migrants without health insurance from seeking necessary testing and treatment.^16^ Stigmatization has also affected Nigerian citizens who are returning to the country from other countries in Europe or Asia. This is highlighted in the case of a Nigerian COVID-19 survivor who reported that her ordeal was made worse by the aura of stigmatization and misinformation that surrounded her during her isolation period. The 29-year-old Nigerian activist was in London, to attend the Commonwealth Day Service, where she was the official flag bearer. She developed symptoms soon after returning.^17^

An online survey conducted in the United States and the United Kingdom showed that participants generally had good knowledge of the main modes of transmission and common symptoms of COVID-19; however, several misconceptions and ideas circulated on social media were held by a substantial proportion of respondents, who also expressed willingness to discriminate against individuals of East Asian ethnicity for fear of contracting COVID-19.^15^ It is worthy of note that higher COVID-19 knowledge scores were found to be significantly associated with a lower likelihood of negative attitudes and potentially dangerous practices toward COVID-19 pandemic as shown in a Chinese survey.^18^ Thus, promoting accurate knowledge of the disease can potentially improve the attitude and practice of the public toward policies designed by government agencies to protect the public health of citizens. There is however a dearth of studies on the knowledge, attitudes, and practices of the Nigerian public concerning the ongoing COVID-19 crisis. This study will fill that gap in knowledge and serve as a reference point for the development of knowledge-based interventions.

The study aimed to assess the knowledge of the cause, mode of transmission, and symptoms of COVID-19, to determine the factors associated with the knowledge of COVID-19; to ascertain the attitudes of the public toward COVID-19; to establish the degree of stigmatization toward foreigners, recent returnees from international travel, and COVID-19 survivors; and to determine the practices associated with COVID-19 among the Nigerian public.

## METHODOLOGY

Nigeria is a vast sovereign territory, made up of 36 states (grouped into six geopolitical zones) and the Federal Capital Territory. It consists of diverse ethnic groups in various regions, with a largely young population.

The study was a cross-sectional survey of the general population of literate Nigerians, who have access to an Internet facility and a computer (mobile phone, laptop, etc.). The study included educated individuals including students (secondary school and university), faculty, and professionals from all walks of life, who understand the English language. There was no age restriction. Those who refused to participate were excluded.

The study was carried out between April and May 2020. The study was questionnaire-based, accessed via an online survey application (Google Forms). The minimum sample size was determined using the Kish formula to be 384,^19^ assuming a confidence level of 95%, absolute precision of 5%, and prevalence of 50%.

A convenience sampling technique was used to recruit interested participants, until the sample size was achieved. The link to the Google Forms questionnaire was disseminated on a daily basis for the duration of one week, via the research collaborators’ social media accounts, and reached a wide audience around the country through rebroadcasting by contacts of the research collaborators. WhatsApp messenger was mainly used in disseminating the link, followed by Facebook, Twitter, LinkedIn, and Instagram.

### Study instrument.

The questionnaire was adapted from a similar study conducted among Chinese residents^18^ and modified to suit the objectives of the study. The questionnaire was pretested and subjected to reliability testing, and a reliability coefficient (Cronbach’s alpha) of 0.82 was obtained. Face validity was established from the pretest, to ascertain that the responses reflected the questions asked and that the questions reflected the study’s objectives. Content validity was established by public health experts who ascertained that the questions explored all important aspects of the topic.

Questions asked in the questionnaire included sociodemographic data, questions on access to information, questions assessing participants’ knowledge of COVID-19 symptoms, transmission and prevention of COVID-19, attitudes toward foreigners of Asian or Caucasian descent, recent returnees from international travel, and COVID-19 survivors, as well as practices relating to the COVID-19 pandemic, including social distancing, handwashing, use of face mask, use of over-the-counter medications, and herbal remedies. Questions on the knowledge section were designed in the true/false pattern, and a scoring system was developed to assess knowledge. The first nine questions were on COVID-19 symptoms and transmission, each allotted one point, whereas the remaining eight questions were on COVID-19 prevention, each allotted two points. The total score was 25 points. All incorrect responses were given no point. A total score (rounded up to the nearest whole number) of less than 13 (< 50%) was graded as poor knowledge, 13–17 (50–69%) intermediate knowledge, and 18–25 (> 70%) good knowledge. The attitude section was designed using a 5-point Likert scale and yes/no questions. Twelve questions were used to score attitude, and each positive attitude was given a score of one point, and a negative attitude was given no point. A total score of < 50% was graded as poor attitude and a total score of > 50% was graded as good attitude. The practice section was designed using yes/no questions and multiple-choice questions. Five questions were used to score practice, and each positive practice was given one point and negative attitudes given no point. A total score of < 50% was graded as poor practice, and a total score of > 50% was graded as good practice.

### Data analysis.

Data collected from the study were collated using Google Sheets (Google LLC, Mountain View, CA) and analyzed using IBM SPSS Statistics for Windows, Version 22.0 (IBM Corp., Armonk, NY). Descriptive statistics were generated. Continuous variables (age, knowledge score, attitude score, and practice score) were transformed into appropriate categories to run the appropriate statistical tests. Student’s *t*-test and analysis of variance (ANOVA) were used to compare continuous variables where appropriate. Tukey’s post hoc test was used to identify the groups within the variables that had statistically significant results with ANOVA. Multivariate analyses were carried out using ordinal logistic regression for knowledge grade (with three categories—good, intermediate, and poor) and binary logistic regression for attitude and practice (with two categories each—good and poor) to determine predictors. The model to obtain predictors of knowledge grade was generated, with odds ratios (OR) and Confidence intervals (CI) reported in the results section; however, models to determine predictors of attitude and practice grade were not significantly different from null models and had a poor fit, suggesting that the relationship between the predictor variables and knowledge grade could not be predicted from our sample, using this method. The independent (predictor) variables in the models include age, gender, occupation, level of education, marital status, and source of information. Marital status was excluded from the final model to improve the model. The level of significance (*P*-value) was set at < 0.05 for all test statistics. All assumptions for the statistical tests used were upheld.

### Ethical considerations.

The aim of the research was explained to all willing participants, and no form of coercion was used in the recruitment process. No identifying information was collected. A statement of informed consent was placed at the beginning of the questionnaire, and only willing participants were to proceed with filling of the questionnaire. The study protocol was approved by the Osun State University Health Research Ethics Committee, with reference number UNIOSUNHREC2020/004.

## RESULTS

The total number of respondents who participated in this study was 1,015. Missing values were stated where present and excluded from the statistical analyses. The ages of the participants ranged from 13 to 65 years, with a mean age of 26.60 ± 7.8 years. There was a slight female preponderance (54.1%). Approximately 90% of the respondents were Christian, and 81.5% were single. More than a third (69.5%) of the respondents had non-medical occupations, whereas 26.6% had medical-related occupations. Most of the respondents (94.4%) had tertiary education. Respondents from the southwest and north central zones accounted for 65% and 18.4% of the sample, respectively, whereas 16.6% were from the other geopolitical zones. Also, more than half of the participants were of the Yoruba ethnic group (62.4%), followed by the Igbos and Hausas (17.2% and 1.8%, respectively). The minority tribes accounted for 18.6% of the population (see Table 1). More than half of the respondents (52.0%) got their updates on COVID-19 from both traditional media (television, radio, and newspaper) and Internet media, whereas 42.6% and 5.4% got their updates on COVID-19 exclusively from Internet media and traditional media, respectively. Nearly all respondents (99.9%) used one form of social media, with WhatsApp (78.1%), Twitter (58%), Instagram (40.7%), and Facebook (35%) being the most popular platforms. More than half of them (51.7%) did not believe that the government is being transparent about the disseminated information concerning COVID-19, whereas 32.6% were unsure.

**Table 1:** Sociodemographics of respondents

	Categories	*N* = 1,015	Percentage
Age-group at last birthday (years)	10–20	147	14.5
	21–30	700	69.0
	31–40	95	9.4
	41–50	44	4.3
	51–60	26	2.6
	61–70	3	0.3
Gender	Female	549	54.1
	Male	466	45.9
Religion	Christianity	909	89.6
	Islam	96	9.5
	Traditional	4	0.4
	Areligious	3	0.3
	Atheism	1	0.1
	Agnostic	1	0.1
	Humanism	1	0.1
Marital status	Single	827	81.5
	Married	181	17.8
	Separated	4	0.4
	Widowed	2	0.2
	Others	1	0.1
Occupation	Medical-related	270	26.6
	Nonmedical-related	705	69.5
	Unemployed	31	3.1
	Missing	9	0.8
Level of education	Tertiary	958	94.4
	Secondary	53	5.2
	Missing	4	0.4
State of residence (geopolitical zones)	Southwest	660	65.0
	Southeast	38	3.7
	South–south	93	9.2
	North central	187	18.4
	Northwest	26	2.6
	Northeast	11	1.1
Ethnicity	Yoruba	633	62.4
	Igbo	175	17.2
	Hausa	18	1.8
	Others	189	18.6

States in the geopolitical zones are as follows: Southwest—Lagos, Ogun, Oyo, Osun, Ondo, and Ekiti; Southeast—Abia, Anambra, Ebonyi, Enugu, and Imo; South–south—Akwa Ibom, Bayelsa, Cross River, and Delta and Edo rivers; North central—Benue, Kogi, Kwara, Nasarawa, Niger, Plateau, and the Federal Capital Territory; Northwest—Jigawa, Kaduna, Kano, Katsina, Kebbi, Sokoto, and Zamfara; Northeast—Adamawa, Bauchi, Borno, Gombe, Taraba, and Yobe.

### Knowledge of COVID-19.

Concerning knowledge of COVID-19, most of the respondents were knowledgeable about the signs and symptoms of the virus as well as the appropriate preventive measures. The most common symptoms that the population knew about were difficulty in breathing (96.9%), dry cough (96%), and fever (93%), with much fewer respondents being aware of the gastrointestinal symptoms (vomiting [17.8%] and diarrhea [19.2%]). More than 90% were aware that there is no cure for the disease and that early conservative treatment would reduce mortality. About half of the respondents felt that only elderly individuals with comorbidities go on to develop severe disease. It is interesting to note that 86.4% of the respondents were not knowledgeable about the fact that asymptomatic carriers can transmit the disease. Most of the respondents were aware of the major preventive strategies for COVID-19, particularly handwashing and social distancing, among others. The knowledge score ranged from 5 to 25, with a mean score of 19.5 ± 2.872, equivalent to a grade of 78% (good knowledge). Using the scoring system, 18 respondents (1.8%) had poor knowledge, 198 respondents (19.5%) had intermediate knowledge, and 799 respondents (78.7%) had good knowledge.

A majority of the respondents who had good knowledge of COVID-19 were single (81.2%), females (55.1%), between the ages of 21 and 30 years (69.2%), and had nonmedical-related occupations (69.8%), and most of them had tertiary education (94.2%) and got their information both from traditional and Internet media sources (52.2%).

There was a significant difference in knowledge scores between age-groups as demonstrated by one-way ANOVA (*F*(5,1009) = 6.310, *P* < 0.001). A Tukey post hoc test showed that the 21- to 30-year age-group (19.82 ± 2.8, *P* < 0.001) had a significantly higher mean knowledge score than the 10- to 20-year age-group (18.66 ± 2.6). There was no statistically significant difference between the other groups. There was a significant difference in knowledge score between occupation groups as demonstrated by one-way ANOVA (*F*(2,1003) = 83.976, *P* < 0.001). A Tukey post hoc test showed that the medical-related occupations (21.30 ± 2.2, *P* < 0.001) had significantly higher mean knowledge scores than the nonmedical-related occupations (18.87 ± 2.8) and the unemployed group (18.42 ± 2.8, *P* < 0.001). There was no statistically significant difference between the other groups (see Table 2).

**Table 2:** Comparing mean COVID-19 knowledge scores between sociodemographic groups of the respondents

Variable	Categories	Knowledge score (mean ± SD)	*t*/*F*-test	*P*-value
Age-group (years)	10–20	18.66 ± 2.6	6.310	**< 0.001**
	21–30	19.82 ± 2.8		
	31–40	19.07 ± 3.2		
	41–50	18.72 ± 3.0		
	51–60	18.37 ± 2.1		
	61–70	19.33 ± 3.4		
Gender	Male	19.39 ± 3.0	−1.121	0.263
	Female	19.59 ± 2.8		
State of residence (geopolitical zones)	Southwest	19.54 ± 2.9	0.724	0.606
	Southeast	18.75 ± 2.8		
	South–south	19.51 ± 2.7		
	North central	19.43 ± 2.7		
	Northwest	19.49 ± 2.9		
	Northeast	20.25 ± 3.7		
Marital status	Single	19.62 ± 2.8	2.243	0.063
	Married	18.96 ± 3.0		
	Separated	18.80 ± 1.2		
	Widowed	18.70 ± 3.0		
	Others	17.40		
Occupation*	Medical-related	21.30 ± 2.2	83.976	**< 0.001**
	Nonmedical-related	18.87 ± 2.8		
	Unemployed	18.42 ± 2.8		
Level of education†	Secondary	19.83 ± 3.1	−0.836	0.404
	Tertiary	19.49 ± 2.9		
Source of information	Traditional media only	19.71 ± 2.7	1.065	0.345
	Internet media only	19.62 ± 2.6		
	Both	19.37 ± 3.0		

*N* = 1,015; level of significance < 0.05. The bold values are the *P*-values that are statistically significant (at *P* < 0.05).

* Occupation, *N* = 1,006.

† Level of education, *N* = 1,011.

It is however worthy of note that respondents with good attitude and practice grades had significantly higher knowledge scores than their counterparts with poor attitude and practice grades (see Table 3). The multiple logistic regression model to determine predictors of knowledge grade was good fitting; it showed that those with medical-related occupations were more likely (OR [95% CI]: 6.559 [2.490–17.277]) to have better knowledge than the unemployed (see Table 4).

**Table 3:** Comparing mean COVID-19 knowledge scores between attitude and practice grades of the respondents

Variable	Categories	Frequency (%)	Knowledge score (mean ± SD)	Student *t*-test	*P*-value
Attitude grade	Poor	93 (9.2)	17.72 ± 3.6	−6.362	**< 0.001**
	Good	922 (90.8)	19.68 ± 2.7		
Practice grade	Poor	248 (24.4)	19.13 ± 2.9	−2.342	**0.019**
	Good	767 (75.6)	19.62 ± 2.9		

*N* = 1,015; level of significance < 0.05. The bold values are the *P*-values that are statistically significant (at *P* < 0.05).

**Table 4:** Logistic regression to determine predictors of good knowledge of COVID-19

Variable	Odds ratio (95% CI)	*P*-value
Age*	0.99 (0.97–1.01)	0.630
Gender		
Male	1.1 (0.8–1.5)	0.679
Female†	–	–
Level of education		
Tertiary	0.8 (0.4–1.6)	0.570
Secondary†	–	–
Occupation		
Medical-related	6.6 (2.5–17.3)	**< 0.001**
Nonmedical-related	1.0 (0.5–2.4)	0.938
Unemployed†	–	–
Source of information		
Traditional media	1.6 (0.8–3.5)	0.204
Internet media	1.2 (0.9–1.7)	0.193
Both†	–	–

The logistic regression model predicts the likelihood that any of the categories of the independent (predictor) variables would have a higher order of COVID-19 knowledge, graded as poor, intermediate, and good knowledge. Level of significance (*P*-value) set at < 0.05. The bold values are the *P*-values that are statistically significant (at *P* < 0.05).

* Age is a continuous variable.

† Reference group within variable.

### Attitudes and perceptions relating to COVID-19.

The vast majority of respondents (97.7%) were certain that COVID-19 exists, and 94% were optimistic that it would be successfully curtailed. Of all the respondents, 78.8% were confident that Nigeria would win the battle against COVID-19. More than three-quarter (80.6%) of them were in support of the self-isolation policy in place to help reduce the spread of the disease. Lack of testing (77.4%) and poor health-seeking behavior (66.5%) were the major perceived reasons for the fewer cases recorded in Nigeria so far; however, some respondents (23.6%) attributed the low number of COVID-19 cases to the hot climate in Nigeria. Regarding the role of China and other foreigners in the emergence of the disease, 65.7% of the respondents blamed China for the pandemic. By contrast, only a minority expressed anger and frustration toward the foreigners in the country, whereas most (77.7%) were indifferent. Over a third of the respondents (37.4%) felt that the disease emerged from eating wild animals, and 44.6% did not believe that previous immunization plays a role in immunity against COVID-19. Of note is the fact that 56.5% of respondents would relate with foreigners, and 82.3% were indifferent about patronizing ventures owned by foreigners, but only 41.9% would interact with Nigerians just returning from international travel. However, most of the respondents (77.5%) would interact with people who had recovered from the disease. The attitude score ranged from 1 to 12, with the mean attitude score being 7.92 ± 1.7 which is equivalent to a grade of 66% (good attitude grade). More than 90% of the participants (922 individuals) had good attitude toward COVID-19.

There was a statistically significant difference in attitude scores between age-groups demonstrated by one-way ANOVA (*F*(5,1009) = 2.597, *P* = 0.024). A Tukey post hoc test showed that the 21- to 30-year age-group (7.98 ± 1.7, *P* = 0.047) had a statistically significantly higher mean attitude score than the 10- to 20-year age-group (7.52 ± 1.9). There was no statistically significant difference between the other groups (see Table 5). There was a statistically significant difference in attitude scores between genders, with males having significantly higher mean scores (*P* < 0.05).

**Table 5:** Comparing mean COVID-19 attitude scores between sociodemographic groups of the respondents

Variable	Categories	Attitude score (mean ± SD)	*t*/*F*-test	*P*-value
Age-group (years)	10–20	7.52 ± 1.9	2.597	**0.024**
	21–30	7.98 ± 1.7		
	31–40	8.05 ± 1.7		
	41–50	7.93 ± 1.9		
	51–60	8.35 ± 1.9		
	61–70	6.33 ± 1.5		
Gender	Male	8.13 ± 1.8	3.522	**< 0.001**
	Female	7.74 ± 1.7		
State of residence (geopolitical zones)	Southwest	7.90 ± 1.7	0.448	0.815
	Southeast	7.63 ± 1.7		
	South–south	8.10 ± 1.8		
	North central	7.97 ± 1.8		
	Northwest	7.96 ± 1.3		
	Northeast	7.82 ± 2.2		
Marital status	Single	7.92 ± 1.8	0.635	0.638
	Married	7.98 ± 1.7		
	Separated	6.75 ± 2.9		
	Widowed	7.00 ± 1.4		
	Others	8.00		
Occupation*	Medical-related	8.30 ± 1.5	8.846	**< 0.001**
	Nonmedical-related	7.78 ± 1.8		
	Unemployed	7.97 ± 1.8		
Level of education†	Secondary	7.66 ± 1.7	1.150	0.250
	Tertiary	7.94 ± 1.7		
Source of information	Traditional media only	7.91 ± 1.6	0.074	0.928
	Internet media only	7.95 ± 1.7		
	Both	7.90 ± 1.8		

*N* = 1,015; level of significance < 0.05. The bold values are the *P*-values that are statistically significant (at *P* < 0.05).

* Occupation, *N* = 1,006.

† Level of education, *N* = 1,011.

There was a statistically significant difference in attitude scores between occupation groups as demonstrated by one-way ANOVA (*F*(2,1003) = 8.846, *P* < 0.001). A Tukey post hoc test showed that the medical-related occupations (8.30 ± 1.5, *P* < 0.001) had a statistically significantly higher mean attitude score than the nonmedical-related occupations (7.78 ± 1.8). There was no statistically significant difference between the other groups.

### Practices relating to COVID-19.

Considering the currently enforced social distancing directives, most of the respondents reported compliance. However, only 22.5% of them reported wearing face masks when leaving home. Considering handwashing as a major preventive measure, up to 90% washed their hands at least twice a day. Although most respondents (95.9%) were aware that there is no cure for COVID-19, 20.1% had taken vitamin supplements, 14.6% home remedies, 2.3% herbal concoctions, 2.2% antibiotics, 1.0% chloroquine and azithromycin, 0.8% chloroquine alone, and 0.5% had taken antiviral medications. The practice score relating to COVID-19 ranged from 0 to 5, with a mean score of 3.02 ± 0.9, which is equivalent to 60.4% (good practice grade). Concerning practices relating to COVID-19, 75.6% (767) of the respondents had good practice. There was no significant difference in mean practice scores among the categories in the various groups (age, gender, occupation, marital status, level of education, state of residence, and source of information).

## DISCUSSION

This cross-sectional study was conducted to determine the knowledge, perceptions, attitudes, and practices of the Nigerian populace in the ongoing COVID-19 pandemic, to fill the paucity of data on the subject matter. In this predominantly well-educated and female population, a significant proportion of our respondents (78.7%) had good knowledge of COVID-19, with an overall knowledge grade of 78%, which is consistent with other population-based studies performed in China and Iran, with overall knowledge scores of 90% each.^18,20^ This high knowledge rate is supported by the fact that 94.4% of the respondents had tertiary education and thus were able to access and comprehend information about the disease, and 65% were from the southwest of the country, which is said to be the geopolitical zone with the highest adult (15+) literacy rate in any language.^21^ Another possible explanation for this finding is that most of the research team members reside in the southwest zone of the country and have most of their Internet contacts from the same zone. Knowledge scores were significantly higher among those aged between 21 and 30 years in our study. This finding may have resulted from overrepresentation of that age-group in the sample, as 69% of the study sample was in that age-group. Those with medical occupations also had significantly higher knowledge scores than the nonmedics and unemployed, although most of them who had nonmedical-related jobs still had considerably good knowledge (> 70%). Our logistic regression model further suggested that respondents with medical-related occupations were likely to have better knowledge than their unemployed counterparts. This differs from the works of Zhong et al.^18^ and Erfani et al.,^20^ who reported that younger age, male gender, being single, and lower level of education were significantly associated with lower knowledge scores.

Furthermore, our study showed that higher knowledge scores regarding COVID-19 were significantly associated with a higher likelihood of having a positive attitude and good practice at the time of the COVID-19 pandemic, with 78.8% of our respondents having confidence that Nigeria will win the battle against COVID-19. This is in agreement with earlier studies.^18,20,22^ This finding of good attitude was especially true for males, those aged 21–30 years, and those in medical-related occupations, as there was a statistically significant association between these characteristics and the mean attitude score. This finding of positive attitude and practice among those with good knowledge strongly suggests that educating the general public about COVID-19 will provide them with the knowledge to enhance their attitude and encourage them to adopt good practices which will ultimately help in reducing disease spread. A majority of the respondents (77.7%) were not overly concerned about the presence of foreigners in relation to the risk of transmission of COVID-19, as 56.5% would still relate with a foreigner in light of the pandemic, although about a third of our respondents (31.8%) strongly agreed that China was responsible for the pandemic. A considerable proportion of our respondents (77.5%) agreed that they would relate with individuals who recently recovered from COVID-19, suggesting that the level of stigma toward them will likely be low in this study population. Most of the respondents with these non-discriminatory attitudes had intermediate-to-good knowledge, although the finding was not statistically significant. Additional studies investigating stigma in the Nigerian populace and around the world toward foreigners, travelers, and COVID-19 survivors would be beneficial, as stigma only generates negative effects on those affected by it.^23^ The use of both traditional and Internet media was the predominant source of information for our respondents, although the knowledge scores were not significantly different from those who used traditional media (television, radio, and newspaper) or Internet media alone. Nearly all our respondents were on social media, with WhatsApp, Twitter, Instagram, and Facebook being the most common platforms for knowledge sharing on COVID-19. Although these platforms are very effective and are used by government and public health agencies to disseminate information on COVID-19, they are also platforms used by rumor-peddlers to spread fear and panic, hence the need for caution by ensuring that information is gotten from verified social media accounts, and the users are also encouraged to go a step further to verify the information from other sources.

Polls conducted in the country when there were two cases of COVID-19 showed 50% were aware of preventive measures, but with Nigeria having 318 cases at the beginning of our study period, majority of participants knew about handwashing, avoiding contact with those with respiratory symptoms, avoiding touching one’s face, and social distancing (99.9%, 97.3%, 98.9%, and 99.3%, respectively).^24,25^ This increase in knowledge could be due to the vast broadcasting and regular Internet and social media updates on preventive measures by the NCDC. This improved knowledge enhanced the behavior as initially only 45% stated that they would practice regular handwashing, but our study showed that 90.6% now reported washing their hands at least twice a day.^24^ In addition, less than a quarter of our sample wore masks on leaving their homes during the study period as it was not recommended for the public, but by April 18, 2020, the NCDC advised the use of masks alongside physical distancing.^26^ Government officials have also recommended the use of improvised face masks by the public.^27^ Although it has been documented that medical face masks reduce viral particles in respiratory droplets,^28^ we recorded low face mask usage in our sample despite the good knowledge of COVID-19. Factors responsible for this may include non-availability or high cost of medical masks, skepticism toward the use of non-medical masks, and worry about what others may think about them, to mention a few. Although the policy to ensure widespread use of face masks in Nigeria may be helpful when combined with other prevention strategies,^29^ there is a need for population studies into the usefulness of non-medical masks in the prevention and/or reduction in community spread of COVID-19.

This study also showed that most of the knowledgeable respondents did not practice self-medication with chloroquine and antibiotics, as well as herbal concoctions, home remedies, and vitamins. Although several non-scientific reports have suggested that the use of herbal preparations has been helpful in the treatment of COVID-19, the authors believe that this is an area that should be given much attention by the scientific community, by way of randomized controlled trials. Although the WHO has launched the “Solidarity” clinical trial for COVID-19 treatments, it recommends that physicians and medical associations avoid recommending the use of unproven treatments to patients with COVID-19 and those self-medicating, except on compassionate basis, where no other alternative exists.^30,31^

Despite an overall knowledge rate of 78%, a large majority of our respondents (86.4%) did not know that COVID-19 patients could present with gastrointestinal symptoms, which can be found in 2–50% of cases,^4^ and that asymptomatic carriers could transmit the disease. We could not find data on the proportion of confirmed cases in Nigeria that were asymptomatic, but some studies elsewhere have highlighted the role of asymptomatic carriers. A retrospective study in Beijing revealed that 5% of the study population were asymptomatic,^32^ and in Japan, the Ministry of Health, Labour, and Welfare announced on March 5, 2020 that among 696 people on a cruise ship that was infected with SARS-CoV-2, 410 were asymptomatic (58.9%).^33^ The Federal Ministry of Health and the NCDC need to pay more attention to disseminating clear and concise information on the atypical symptomatology of the disease, and emphasizing the role of “well” carriers in the transmission of COVID-19 in their health education broadcasts.

This study was not without its limitations. To reduce the risk of transmission of COVID-19, our study was web based not community based. However, the Digital 2020 Global Overview Report revealed that Internet penetration in Nigeria is only 42%.^34^ Besides, we had an overrepresentation of individuals with tertiary level of education, despite the country’s adult (15+) literacy rate being 62%.^35^ Therefore, these results may not be representative of the entire Nigerian population, as the uneducated and those without access to the Internet are unaccounted for. This study used a consecutive (convenience) sampling technique because of movement restrictions in place during the sampling period, which is a non-probability sampling method. Future studies that would use a probability sampling technique and also include people who do not have access to the Internet (including the uneducated) would be beneficial to validate our results, as these people can potentially drive the infection locally.

## CONCLUSION

This Nigerian sample was mostly made up of young, well-educated, female respondents, with nonmedical careers, with the highest knowledge demonstrated among those aged 21–30 years, females, and those in medical professions. Nearly all of our respondents used social media and got their updates on COVID-19 mostly through WhatsApp, Twitter, Instagram, and Facebook. The majority exhibited good knowledge of COVID-19, as well as positive, non-stigmatizing perceptions, and attitudes toward COVID-19, foreigners (particularly of Asian and Caucasian descent), recent Nigerian returnees from international travel, and COVID-19 survivors. Social distancing and handwashing were reported to be well adhered to and most respondents did not practice self-medication. It is recommended that public health education campaigns continue, by engaging the public more on social media and other Internet platforms, to reach the younger population, and other traditional media to reach the older generation—providing updates on clinical presentation, prevention, and control strategies; evidence-based policies; and debunking of myths. This would reduce stigma to groups at risk, improve attitude in general, and also improve adherence to preventive practices. There is need for further studies of other groups within Nigeria, such as the less educated, more rural, and those without Internet access, to get a clearer picture of the Nigerian situation as regards COVID-19 knowledge, attitudes, and practices, and proffer appropriate interventions. There is also a need for local and international collaborative research efforts on COVID-19 treatment and prevention options to bring a rapid end to the ongoing crisis.
